# Common diagnoses among pediatric attendances at emergency departments

**DOI:** 10.1186/s12887-021-02646-8

**Published:** 2021-04-14

**Authors:** Shuen Yin Celine Yoong, Peck Har Ang, Shu-Ling Chong, Yong-Kwang Gene Ong, Nur Diana Bte Zakaria, Khai Pin Lee, Jen Heng Pek

**Affiliations:** 1 Yong Loo Lin School of Medicine, National University of Singapore, 10 Medical Dr, Singapore, 117597 Singapore; 2 Accident and Emergency Department, Changi General Hospital, 2 Simei Street 3, Singapore, 529889 Singapore; 3 Department of Emergency Medicine, KK Women’s and Children’s Hospital, 100 Bukit Timah Rd, Singapore, 229899 Singapore; 4 Department of Emergency Medicine, Singapore General Hospital, Outram Rd, Singapore, 169608 Singapore; 5 Department of Emergency Medicine, Sengkang General Hospital, 110 Sengkang E Way, Singapore, 544886 Singapore

**Keywords:** Attendance, Diagnosis, Emergency, Health services, Pediatric

## Abstract

**Background:**

Pediatric patients present to Emergency Departments (EDs) with a variety of medical conditions. An appreciation of the common presenting conditions can aid EDs in the provision of pediatric emergency care. In this study, we established the common pediatric diagnoses seen at the general EDs, with reference to a pediatric ED.

**Methods:**

A retrospective review of medical records was performed for patients less than 16 years old at a pediatric ED and two general EDs from 1 January to 31 December 2018. Information including patient demographics, triage category, case type and diagnoses were collected.

**Results:**

There were 159,040 pediatric attendances, of which 3477 (2.2%) were seen at the general EDs. Non-traumatic conditions were most prevalent at both general (*N* = 1933, 55.6%) and pediatric (*N* = 128,415, 82.5%) EDs. There was a higher proportion of trauma related conditions seen at the general EDs (*N* = 1544, 44.4%) compared to the pediatric ED (*N* = 27,148, 17.5%; *p* < 0.01). Across all EDs, upper respiratory tract infection, unspecified musculoskeletal pain and gastroenteritis were the three most common non-trauma related diagnoses, while fracture, wound and contusion were the three most common trauma related diagnoses. There was a greater proportion of emergent (P1) cases seen at the general EDs (*N* = 233, 6.7%) than the pediatric ED (*N* = 3821, 2.5%; *p* < 0.01). Respiratory conditions including bronchiolitis, asthma and bronchitis were the most common emergent (P1) diagnoses.

**Conclusions:**

The common diagnoses among pediatric attendances varied between pediatric and general EDs. Therefore, general EDs should focus their efforts on these common diagnoses, especially the emergent (P1) ones, so that they can enhance their preparedness and work towards providing quality pediatric emergency care.

## Background

Utilization of Emergency Departments (EDs) by pediatric patients has increased over the years [1]. Pediatric patients differ from adult patients in terms of their anatomy, physiology, development and emotional needs. Pediatric patients also present with a different spectrum of clinical ailments compared to their adult counterparts [2].

General and pediatric EDs have varying capabilities in the provision of pediatric emergency care [3–7]. Pediatric EDs are staffed by pediatric emergency physicians who are able to address the unique clinical and psychosocial needs of children and their families [3, 8]. On the other hand, general EDs are staffed with emergency physicians with variable pediatric experiences. Reduced familiarity with pediatric conditions and their management can lead to unnecessary workup in the ED, leading to a strain on limited resources, longer length of stay, and increased rates of reattendance, admission and mortality [9, 10]. It is imperative that emergency physicians remain familiar with the management of pediatric conditions so as to ensure that the safety and quality of pediatric emergency care is not compromised in general EDs [8].

Understanding the needs of children who attend the EDs can inform improvement of pediatric emergency care via the implementation of clinical guidelines and workflows, acquisition of suitable manpower and equipment, and provision of holistic training to ED staff. Given the breadth of pediatric conditions presenting to EDs, identifying the common diagnoses can allow EDs to focus and maximize their efforts on essential and high-yield services [11]. This study aimed to establish the common diagnoses among pediatric patients at the general EDs, with reference to a pediatric ED.

## Methods

### Study setting

Singapore is an urban city with a land area of 728 km^2^ and a population of 5686800 [12]. Persons under the age of 20 years old make up 14.1% of the population. The healthcare system consists of private and public institutions. At the nine public acute hospitals where more than 80% of all hospital beds are located, healthcare for Singapore citizens and permanent residents are provided at a subsidised rate with up to 80% of the costs covered by the government. These public hospitals are grouped into three healthcare clusters based on geographical locations.

This study was carried out in a pediatric tertiary hospital and two adult tertiary hospitals which belong to the same healthcare cluster. The 830-bed pediatric hospital has an ED with an annual census of 142,000. It is supported by inpatient and outpatient pediatric specialties. The two adult hospitals, one 1000- and another 1700-bed hospital, have general EDs with annual census of 157,000 and 165,000 respectively. There are no pediatricians in these EDs and the hospitals have no pediatric inpatient units. The distances between these three hospitals range from 4 to 20 km.

Primary care for pediatric patients is provided at about 1700 clinics run by private general practitioners and 20 polyclinics which are ‘one-stop’ healthcare centers offering primary care services owned by the government. Onward referral to the acute hospitals may be made by the primary care providers for urgent conditions. However, primary care consult is not required before a patient can attend the ED of an acute hospital.

Pre-hospital emergency medical services are provided by a single national provider, the Singapore Civil Defence Force. When activated, patients will be conveyed to the public hospitals. Pediatric patients less than 16 years old will be conveyed to the pediatric EDs. However, if they are critically ill with imminent airway compromise, profound shock or cardiopulmonary arrest, they will be conveyed to the nearest ED for assessment and stabilization, regardless of whether it is a general or pediatric ED. After stabilization, the patients will then be transported to the public pediatric hospitals by either the ED team or the pediatric hospital’s transport team for further management.

### Study design

In this retrospective study, all patients less than 16 years old who attended the three EDs in the healthcare cluster between 1 January to 31 December 2018 were included. Their electronic medical records were accessed and the following data were extracted: patient demographics, triage category, case type (trauma or non-trauma) and SNOMED Clinical Terms diagnoses codes.

This study was approved by Institutional Review Board at SingHealth, Singapore (CIRB reference 2019/2360), with waiver of informed consent. All methods were carried out in accordance with relevant guidelines and regulations.

### Statistical methods

SPSS version 25 (SPSS, Chicago, IL) was used to perform the statistical analysis. Categorical data were presented as frequencies with percentages. Continuous data were presented as median with interquartile range (IQR). Association between categorical variable was assessed using chi-square test. Statistical significance was taken at *p* < 0.05.

## Results

### Characteristics of pediatric attendances

There were 159,040 pediatric attendances during the study period, of which 3477 (2.2%) were seen at the general EDs and 155,563 (97.8%) were seen at the pediatric ED (Table 1). The median age of pediatric patients seen in the general EDs (11 years, IQR 5 to 14) was higher than those seen in the pediatric ED (3 years, IQR 1 to 7). There was a greater proportion of emergent (P1) cases at general EDs (*N* = 233, 6.7%) than the pediatric ED (*N* = 3821, 2.5%; *p* < 0.01). Non-traumatic conditions were most prevalent in both general (*N* = 1933, 55.6%) and pediatric EDs (*N* = 128,415, 82.5%). However, there was a greater proportion of trauma related conditions at the general EDs (*N* = 1544, 44.4%) than the pediatric ED (*N* = 27,148, 17.5%; *p* < 0.01).

**Table 1:** Characteristics of pediatric attendances at the emergency departments

	General ED A	General ED B	Pediatric ED
**Pediatric Attendances, N**	2326	1151	155,563
**Proportion of Pediatric Attendances (%)**	1.5	0.7	97.8
**Age (Years), Median (IQR)**	11 (6 to 14)	9 (4 to 13)	3 (1 to 7)
**Gender, N (%)**			
**Male**	1368 (58.8)	634 (55.1)	86,700 (55.7)
**Triage Category**^**a**^**, N (%)**			
**Emergent (P1)**	139 (6.0)	94 (8.2)	3821 (2.5)
**Urgent (P2)**	868 (37.3)	737 (64.0)	82,402 (53.0)
**Ambulatory (P3)**	1319 (56.7)	320 (27.8)	69,340 (44.5)
**Case Type, N (%)**			
**Non-trauma**	1150 (49.4)	783 (68.0)	128,415 (82.5)
**Trauma**	1176 (50.6)	368 (32.0)	27,148 (17.5)

^a^Patients in the P1 category are critically ill and require immediate medical attention and resuscitation. Patients in the P2 category are not in imminent danger of cardiovascular collapse but are in some form of distress, therefore requiring early medical attention and urgent intervention. Patients in the P3 category have mild to moderate symptoms and do not require early medical attention or urgent intervention

### Common diagnoses

Three out of the five most common diagnoses were non-trauma related diagnoses at the general EDs while all five were non-trauma related diagnoses at the pediatric ED (Table 2). At the general EDs, the five most common diagnoses accounted for 47.2 and 39.4% of the pediatric attendances (Table 3). In comparison, the five most common diagnoses accounted for 44.0% of the attendances at the pediatric ED.

**Table 2:** Five most common diagnoses seen at the general (combined) and pediatric emergency departments

	Diagnosis	N (%)	Median Age in Years (IQR)
**General EDs (Combined) (*N*** **= 3477**^**a**^**)**	1) Wound (laceration/abrasion)	419 (12.1)	7 (4 to 12)
	2) Fracture	401 (11.5)	12 (9 to 14)
	3) Upper respiratory tract infection	330 (9.5)	8.5 (3 to 13)
	4) Unspecified musculoskeletal pain	177 (5.1)	13 (11 to 14)
	5) Gastroenteritis	176 (5.1)	12 (6 to 14)
**Pediatric ED (*N*** **= 155,563)**	1) Upper respiratory tract infection	31,013 (19.9)	2 (1 to 5)
	2) Unspecified fever	11,114 (7.1)	2 (0 to 4)
	3) Gastroenteritis	10,151 (6.5)	2 (1 to 6)
	4) Pneumonia	10,059 (6.5)	3 (1 to 5)
	5) Unspecified gastrointestinal symptoms	6069 (3.9)	5 (2 to 9)

^a^Missing data: 17 (0.3%)

**Table 3:** Five most common diagnoses seen at the general emergency departments

	Diagnosis	N (%)
**General ED A (*N*** **= 2326**^**a**^**)**	1) Fracture	320 (13.8)
	2) Wound (laceration/abrasion)	319 (13.7)
	3) Upper respiratory tract infection	178 (7.7)
	4) Contusion	143 (6.1)
	5) Unspecified musculoskeletal pain	138 (5.9)
**General ED B (*N*** **= 1151**^b^**)**	1) Upper respiratory tract infection	152 (13.2)
	2) Wound (laceration/abrasion)	100 (8.7)
	3) Fracture	81 (7.0)
	4) Unspecified fever	66 (5.7)
	5) Gastroenteritis	54 (4.7)

^a^Missing data: 1 (0.04%)

^b^Missing data:16 (1.4%)

For non-trauma related diagnoses, upper respiratory tract infection, (URTI) unspecified musculoskeletal pain and gastroenteritis were the three most common diagnoses seen at the general EDs (*N* = 330, 9.5%; *N* = 177, 5.1%, *N* = 176, 5.1% respectively), while URTI, unspecified fever and gastroenteritis were the three most common diagnoses seen at the pediatric ED (*N* = 31,013, 19.9%; *N* = 11,114, 7.1%; *N* = 10,151, 6.5% respectively). For trauma related diagnoses, wound, fracture and contusion were the three most common diagnoses seen at the general EDs (*N* = 419, 12.1%; *N* = 401, 11.5%; *N* = 173, 5.0% respectively), while fracture, wound and head injury were the three most common diagnoses seen at the pediatric ED (*N* = 5756, 3.7%; *N* = 5350, 3.4%; *N* = 3776, 2.4% respectively).

Among the critically ill pediatric patients in the emergent (P1) category, febrile (*N* = 41, 17.6%) and non-febrile (*N* = 27, 11.6%) seizure disorders, as well as allergic conditions (*N* = 15, 6.4%) were the three most common emergent (P1) diagnoses at the general EDs, while bronchiolitis (*N* = 452, 11.8%), asthma (*N* = 417, 10.9%), and bronchitis (*N* = 320, 8.4%) were the three most common emergent (P1) diagnoses seen at the pediatric ED (Table 4).

**Table 4:** Top 5 diagnoses among emergent (P1) patients in pediatric and general emergency departments

	Diagnosis	N (%)	Median Age in Years (IQR)
**Pediatric ED (*N*** **= 3821)**	1) Bronchiolitis	452 (11.8)	0 (0 to 1)
	2) Asthma	417 (10.9)	4 (2 to 6)
	3) Bronchitis	320 (8.4)	3 (2 to 4)
	4) Unspecified fever	244 (6.4)	2.5 (0 to 6.75)
	5) Pneumonia	233 (6.1)	2 (1 to 4)
**General ED (*N*** **= 233)**	1) Febrile seizure	41 (17.6)	2 (1 to 3)
	2) Non-febrile seizure	27 (11.6)	2 (1 to 9)
	3) Allergic conditions	15 (6.4)	13 (1 to 15)
	4) Cardiac arrest	15 (6.4)	6 (3 to 10)
	5) Burns	12 (5.2)	3.5 (1 to 7.5)

## Discussion

The five most common diagnoses represented majority of pediatric attendances at the EDs. While non-trauma related conditions were more common across all EDs, the proportion of trauma related diagnoses was higher in general EDs. General EDs saw a greater proportion of pediatric patients with emergent (P1) conditions, of which seizure disorders were most commonly seen. Respiratory conditions were the most common emergent (P1) condition seen at the pediatric ED. Insights gleaned from this work can be used to tailor emergency services to the specific needs of children and their families.

Firstly, a distinct pattern of emergency service utilization by pediatric patients was identified. Similar to studies in the United Kingdom where respiratory, febrile and gastrointestinal complaints were most commonly encountered in pediatric EDs [13, 14], URTI, unspecified fever and gastroenteritis were most commonly seen in pediatric ED in Singapore. The top five diagnoses at the pediatric ED were also exclusively non-trauma related. This contrasts the common diagnoses seen in adult patients at general EDs, where unspecified abdominal pain, musculoskeletal strain/sprain, and contusions have been reported to be most frequent [15]. Pediatric patients have discrete medical conditions with specific management priorities which differ from adult patients. Hence, it is important for EDs to develop guidelines, pathways, and training unique to the pediatric population to optimize pediatric emergency care.

Secondly, general EDs should focus their attention on the common emergent (P1) diagnoses among critically ill children due to the urgency of such cases. There was also a higher proportion of these cases at the general EDs than the pediatric ED which was attributable to the need for conveyance to the nearest ED for timely assessment and stabilization, and general EDs being more likely to triage pediatric patients to higher acuity due to inadequate training, experience and familiarity with pediatric patients and their clinical conditions [16]. Therefore, prioritizing the care of emergent (P1) cases will increase the preparedness of EDs, leading to accurate decisions and swift interventions during resuscitation. In addition to developing resuscitation algorithms, emergency team training via simulation sessions can be conducted based on these common conditions. Rotating emergency teams from general to pediatric EDs can help increase exposure to pediatric cases, thereby enhancing the quality and safety of pediatric emergency care in general EDs [17].

Next, the common diagnoses differed between pediatric and general EDs. Similar to studies down in the United States, general EDs in Singapore are more likely to encounter injuries and musculoskeletal complaints due to the sudden and distressing nature of traumatic injuries [9]. When a child presents with acute gross deformities or a profuse bleed, caregivers may choose to bring their child to the nearest ED rather than travel further to a pediatric ED due to the perceived urgency of the condition. Caregivers may also have differing expectations of the care provided by different EDs, of which traumatic complaints could be satisfactorily addressed in most EDs, while non-traumatic complaints require the expertise and reassurance of pediatric specialists [10, 18]. Thus, it is important that general ED physicians have the competence and confidence in managing trauma cases unique to the pediatric population. Additional considerations such as the use of computed tomography in pediatric head injury, and the importance of sedation and analgesia in pediatric wound management must not be ignored.

Lastly, the common diagnoses across all EDs reflect the healthcare-seeking behaviours of pediatric patients and their caregivers. This healthcare-seeking behaviour may be fuelled by the healthcare system in Singapore as there are only two pediatric EDs in Singapore where care is provided at a subsided rate and attendance to the ED does not require primary care referral. Regardless, studies have shown that caregivers have a predilection for seeking pediatric care at EDs, even if their conditions do not necessitate emergent medical attention [2, 9, 19]. Similarly, in this study, some common pediatric diagnoses seen at EDs, such as URTI, gastroenteritis and unspecified fever, can be adequately addressed by primary care providers. The inclination to seek medical help at EDs may be due to round-the-clock accessibility, perceived higher quality of care, and greater patient/caregiver satisfaction at EDs [20]. There is a need to reshape the delivery of primary care in Singapore such that pediatric patients seek care at appropriate medical facilities that are best tailored for their condition. Caregiver education on pediatric health and ED utilization can also reduce unnecessary ED visits for non-urgent conditions, thereby minimising the strain on limited resources. Inefficiencies and delayed care for critically ill patients in EDs can thus be reduced [21, 22].

### Limitations

This retrospective study involved pediatric attendances at the EDs over a 1 year period, resulting in a limited sample size and any trends over time in the common diagnoses could not be established. We did not present collective data on the common diagnoses across all EDs in the healthcare cluster as the number of patients at the pediatric ED was significantly larger than the general EDs and this could skew the data leading to misrepresentation. Using patients’ electronic medical records, the diagnoses were captured based on the SNOMED Clinical Terms diagnoses codes entered by the doctor during consultation. The accuracy of the diagnoses was not verified against the clinical details of the consult due to missing information and inconsistencies in documentation by various medical personnel. We did not analyse the initial presenting complaints because these were entered as free text in our hospitals due to the lack of a widely accepted categorization system [23]. We also did not ascertain the reasons behind the observed pattern of ED utilization by paediatric patients. This study was carried out in a single healthcare cluster consisting of only tertiary hospitals linked to academic centres in urban areas. The common diagnoses identified may differ in other hospitals such as community and non-academic centres, and settings such as those with differing population density and without a pediatric ED located nearby. A collaborative effort at an international or national level can be built upon this work to allow for a more in-depth understanding of the common diagnoses among pediatric patients at EDs, and how it varies across healthcare institutions and settings. This knowledge can help reshape and define the role of EDs in its delivery of pediatric care.

## Conclusions

Understanding the common diagnoses among children presenting to EDs can facilitate competency-training and capacity-building for optimal delivery of pediatric emergency care. Focusing on these high-yield and crucial areas of need amongst pediatric patients can achieve maximal impact on preparedness. EDs need to ensure that management guidelines, healthcare provider training and resources are adequate and able to deliver high quality care to children across all EDs.

## Data Availability

The datasets generated and analyzed during the current study are not publicly available as per guidelines by Institutional Review Board at SingHealth, Singapore but may be available from the corresponding author on reasonable request.
